# Polymorphisms in MicroRNA Target Sites of Forkhead Box O Genes Are Associated with Hepatocellular Carcinoma

**DOI:** 10.1371/journal.pone.0119210

**Published:** 2015-03-04

**Authors:** Chao Tan, Shun Liu, Shengkui Tan, Xiaoyun Zeng, Hongping Yu, Anhua Li, Chunhua Bei, Xiaoqiang Qiu

**Affiliations:** 1 Department of Epidemiology, School of Public Health, Guangxi Medical University, Nanning, Guangxi, China; 2 Department of Epidemiology, School of Public Health, Guilin Medical University, Guilin, Guangxi, China; Sanjay Gandhi Medical Institute, INDIA

## Abstract

The forkhead box O (FOXO) transcription factors play important roles in various cancer development including Hepatocellular Carcinoma (HCC). In this study we conducted a hospital-based case control study including 1049 cases (HCC patients) and 1052 controls (non-tumor patients) to examine whether single nucleotide polymorphisms (SNPs) within microRNA (miRNA) target sites of *FOXO* genes confer HCC susceptibility. A total of three miRNA target site SNPs in the 3’ untranslated regions (UTR) of *FOXO1* (rs17592236), *FOXO3* (rs4946936) and *FOXO4* (rs4503258) were analyzed. No statistically significant differences were found in genotype distribution for rs17592236, rs4946936, and rs4503258 between the HCC patient group and the tumor-free control group using single factor chi-square analysis (*P*>0.05). However, multivariate logistic regression analysis showed that the CT/TT genotype in rs17592236 was significantly associated with decreased risk of HCC development (*P* = 0.010, OR = 0.699, 95% CI: 0.526–0.927) as compared to the CC genotype in rs17592236. Additionally, a genetic interaction was found between rs17592236 and rs4503258 (*P* = 0.003, OR = 0.755, 95% CI: 0.628–0.908). Functional dual luciferase reporter assays verified that the rs17592236 SNP was a target site of human miRNA miR-137. Together, these results indicate that the rs17592236 polymorphism is associated with decreasing of HCC hereditary susceptibility likely through modulating the binding affinity of miR-137 to the 3’UTR in *FOXO1* messenger RNA (mRNA). Further knowledge obtained from this study may provide important evidence for the prevention and targeted therapy of HCC.

## Introduction

Hepatocellular Carcinoma (HCC) is a complicated disease caused by a combination of both genetic and environmental factors [1]. However, the etiology underlying HCC has not yet been illustrated. The forkhead box O (*FOXO*) transcription factors constitute a subfamily of forkhead box (*FOX*) transcription factors that, in humans, include *FOXO1*, *FOXO3*, *FOXO4* and *FOXO6* [2]. These factors play important roles in cellular metabolism, transformation, survival and proliferation [3–5]through complex pathways [6]. Previous studies have shown that *FOXO* transcription factors serve as important tumor suppressors [7–9]. Additionally, recent studies have indicated that abnormal expression of *FOXO* genes is associated with the development of human tumors [10, 11], as well as cancers including HCC [12–15].

Single nucleotide polymorphisms (SNPs) are the most frequent variations in the human genome and those residing in non-coding control regions have been recognized as promising candidates for regulation of gene expression. MicroRNAs (MiRNA) are non-coding RNAs consisting of approximately 20–24 nucleotide base-pairs that target genes within their 3’untranslated region (3’UTR) of mRNA. Binding to the target site leads to cleavage of target mRNA and/or repression of mRNA translation. Emerging evidence suggest that 3’UTR SNPs located within miRNA target sites are associated with carcinogenesis,which is likely the result of altered miRNA binding to target mRNAs[16, 17]. In this study, we entertain the hypothesis that misregulation of FOXO gene expression through SNPs in miRNA target sites is associated with HCC development. We selected three miRNA target site SNPs from the NIEHS database (http://snpinfo.niehs.nih.gov/) for this study: rs17592236 (C>T), rs4946936 (C>T) and rs4503258 (C>T) which are SNPs located in the 3’UTR of *FOXO1*, *FOXO3* and *FOXO4*, respectively [18, 19]. A survey with the microRNA database (http://www.microrna.org/) [20] revealed that all three SNPs are located within potential miRNA target sites: rs17592236 (miR-137), rs4946936 (miR-1182), rs4503258 (miR-1322). In light of these facts, we hypothesize that sequence variations at the three selected SNPs may either increase or decrease the risk of HCC by modulating miRNA target binding affinity. To test this hypothesis, we conducted a case-controll study examining whether these three SNPs were associated with risk of HCC development. Subsequently, luciferase function assays were conducted in order to clarify the potential mechanism.

## Materials and Methods

### Patient Subjects

Our study was designed as a hospital-based case control study. Before participation, patient subjects received a detailed description of the study protocol and signed informed consent. The study protocol and the consent forms were approved by the institutional review board of the Tumor Hospital of Guangxi Medical University and the First Affiliated Hospital of Guangxi Medical University. All patients were recruited from June 2007 to April 2011 in the Tumor Hospital of Guangxi Medical University. Cases were selected by histo-pathology of newly diagnosed HCC patients. Controls were collected from the First Affiliated Hospital of Guangxi Medical University’s non-tumor patients within the same period of time. Participation rates were 85% and 78% for HCC patients and the controls, respectively. The overall participation rate for all participants was 81%. Among the subjects, 589 HCC cases and 597 controls were collected between June 2007 and January 2010 as reported in our earlier studies [21, 22]. Hence, an additional 460 HCC cases and 455 controls were recruited from February 2010 to April 2011. Altogether, a total of 1049 HCC cases and 1052 controls were used in the present study. All subjects were all born in Guangxi, and frequency matched by age, gender, and ethnicity.

### Sample Collection and Questionnaire Survey

Two mL of peripheral blood was collected into a vacuum EDTA anticoagulant tube before receiving therapy. Afterwards, genomic DNA was extracted from the blood and stored at-80℃. The sample collection process included face-to-face interviews conducted by trained investigators. The interviews were based on epidemiological investigation questionnaires that covered basic information, smoking habits, alcohol intake, chronic HBV infection, HCC family history, etc.

## SNP Screening

SNP screening of *FOXO1*, *FOXO3*, *FOXO4*, and *FOXO6* was conducted using the NIEHS database (http://snpinfo.niehs.nih.gov/). SNPs were screened with primary restricted conditions of minor allele frequency (MAF) in Chinese population >0.05 and being potential target sites of miRNA. Using these criteria, rs17592236 (C>T) of *FOXO1*, rs4946936 (C>T) of *FOXO3* and rs4503258 (C>T) of *FOXO4* were selected for further study. No SNP in the *FOXO6* gene meets the above two criteria.

### Genotyping

The TaqMan MGB real-time fluorescent quantitative PCR technology was used for genotyping following manufacturer’s instruction. Genotyping was performed in a 7500 Fast (ABI) real-time qPCR machine with the TaqMan SNP genotyping kit containing 2×TaqMan Universal PCR Master Mix (ABI) and 20×TaqMan SNP Genotyping Assay Mix (ABI). PCR reactions (10μL) consisted of 5μL 2×TaqMan Universal PCR Master Mix, 0.25μL 20×SNP Genotyping Assay Mix, 4.35μL ddH2O, and 0.4μL DNA sample(1~10ng/μL). PCR reactions were carried out as: 95℃ × 10min→(95°C×15s→60°C×1min)× 50 cycles. Two blank control wells were used in each 96-well plate. The results of the genotyping were analyzed with the 7500 Fast System V1.3.1 SDS software.

### Vector Construction

To create a DNA construct carrying the rs17592236 wild type *C* allele (*FOXO1*-wild), a 3385bp fragment of the *FOXO1* 3’UTR was amplified from human genomic DNA and cloned into the miRNA Expression Reporter Vector(pMIR-REPORT miRNA Expression Reporter Vector System,Invitrogen)The primer sequences were: F 5’GACTAGTCCTTGTGGCTGACAAGACTTAACTCAAGTATT 3’ and R 5’ CCGAGCTCGGGTTAGTGAGCAGGTTACACTT 3’. Next, a mutant *T* type allele *(FOXO1*-mut) of rs17592236 was similarly constructed. The primer sequences were: F 5’CCTCGTTTGACAAAGGATCATTGCTTTAGATG 3’ and R 5’ATCCTTTGTCAAACGAGGGATTTTGATCCAC 3’. All constructs were confirmed by sequencing.

### Cell Culture and Co-transfection

293T (human embryonic kidney cell line) cells were purchased from the Chinese Academy of Sciences cell bank (Shanghai) and cultured in Dulbecco’s modified Eagle’s medium (Gibco) supplemented with 10% Fetal Bovine Serum (Gibco) and 2mM L-glutamine (Gibco) at 37°C with 5% Carbon Dioxide (CO_2_). Low passage cells were used in experiments. 24 hours before transfection, cells were plated onto a 96-well plate at a density of 1×10^5^ cells/well. Cells were plated in 5 groups that each contained 6 wells: Group 1[(*FOXO1*-wild plasmids and hsa-miR-137 miRNA mimics (Ambion)], Group 2 (*FOXO1*-mut plasmids and hsa-miR-137 miRNA mimics), Group 3 (blank plasmids and hsa-miR-137 miRNA mimics), Group 4 (only transfection reagents), and Group 5 (293T cells without transfection). When cells reached 80% confluence, they were transiently co-transfected with reporter plasmids and hsa-mir-137 mimics (Ambion) using Lipofectamine 2000 (Invitrogen) following manufacturer’s instructions. The PMIR-REPORT β-galactosidase control plasmid was used to normalize transfection efficiency.

### Dual-Luciferase Assays

Cells were harvested for luciferase assays 24 hours after transfection, using the Dual-Light Luciferase & β-Galactosidase Reporter Gene Assay System (ABI) with a MPL1 Economical Microplate Luminometer (Berthold, Germany) following manufacturer’s instructions. All assays were in triplicates and repeated three times.

### Statistical Analysis

The EpiData3.1 software was used for data entry and consistency check, while the SPSS 20.0 software was used for statistical analyses. More specifically, statistical analyses were performed using t-test and chi-square test. Adjusted logistic regression models were used to calculate odds ratio (OR) and 95% confidence interval (CI). Gene-environment interactions and gene-gene interactions were performed through logistic regression models to evaluate multiplicative interaction. Each interaction (a SNP×an environment factor, a SNP×another SNP) was evaluated one at a time in this model. In the Gene-Environment and Gene-Gene Interaction, the three SNPs were all modeled as CC versus CT/TT. Reference category of each SNP is 1 = CC. The environmental factors were modeled as 0 = No, 1 = Yes, and reference category is 0 = No. Covariates included in the logistic regression models were parameters that may be involved in the progression of HCC. Covariates included gender (male or female), age (years), ethnicity (Han, Zhuang, and Others), smoking (yes, no), alcohol intake (yes, no), chronic HBV infection (yes, no), family history of HCC (yes, no). “Smoking” and “Alcohol intake” indicate that a participant is an ever smoker/ drinker. The information on “Chronic HBV infection” was obtained from the patient’s medical record and confirmed by laboratory tests. “Family history of HCC” means one or more direct blood relatives of a participant have suffered from HCC. Hardy-Weinberg Equilibrium (HWE) in the controls was tested using the Haploview 4.2 software.

## Results

### Characteristics of the participants

The general categorization of the participants is presented in Table 1. No statistically significant difference with regard to age, gender, and ethnicity was found between case and control populations (*P* >0.05). However, the percentage of subjects that responded positively to smoking, alcohol intake, chronic Hepatitis B Virus (HBV) infection, and HCC family history was significantly higher among the HCC cases than controls (*P* <0.05).

**Table 1:** Distributions of general demographic characteristics and environmental risk factors in the cases and the controls.

Characteristics	Cases(n = 1049)	%	Controls(n = 1052)	%	*P*-value
Age(years, $\bar{x}\pm s$)	48.30±11.02		47.47±11.80		0.10
Gender					0.16
Male	911	86.84	891	84.70	
Female	138	13.16	161	15.30	
Nationalities					0.19
Han	709	67.59	673	63.97	
Zhuang	322	30.70	362	34.41	
Others	18	1.72	17	1.62	
Smoking					<0.01
Yes	399	38.04	168	15.97	
No	650	61.96	884	84.03	
Alcohol intake					<0.01
Yes	369	35.18	154	85.36	
No	680	64.82	898	14.64	
Chronic HBV infection					<0.01
Yes	877	83.60	130	12.36	
No	172	16.40	922	87.64	
Family history of HCC					<0.01
Yes	46	4.39	7	0.67	
No	1003	95.61	1045	99.33	

### Distribution of Genotype Frequency and Risk of HCC

The genotype frequencies of rs17592236 and rs4946936 were in line with Hardy-Weinberg equilibrium (HWE) in the controls (tested with HaploView4.2). The HWE test was not performed on *FOXO4* rs4503258 due to the fact that it is located on the X-chromosome. The genotype distributions of the three SNPs are shown in Table 2. No statistically significant differences in genotype frequency distributions of the SNPs was detected between the HCC cases and controls using the single factor chi-square analysis (*χ* ^*2*^ = 1.12, *P* = 0.572 for rs17592236; *χ* ^*2*^ = 3.21, *P* = 0.201 for rs4946936; *χ* ^*2*^ = 3.06, *P* = 0.216 for rs4503258). A logistic regression analysis was performed in order to adjust for the potential demographic and environmental differences among the cases and controls. Results from this analysis indicated that individuals carrying the rs17592236 mutant allele T (CT/TT) had a decreased risk of HCC as compared to those with the CC genotype. However, no significant correlation was observed between the other two SNPs and HCC risk.

**Table 2:** Associations between candidate SNPs and HCC.

Genotypes	Cases [n (%)]		Controls [n (%)]		OR (95%CI)^a^	*P*-value^a^	OR (95% CI)^b^	*P*-value^b^
rs17592236								
CC	356	33.94	348	33.08	1.00		1.00	
CT	510	48.62	534	50.76	0.93(0.77–1.13)	0.48	0.70(0.53–0.93)	0.01
TT	183	17.45	170	16.16	1.05(0.82–1.36)	0.70	0.72(0.49–1.05)	0.09
CT/TT	693	66.06	704	66.92	0.96(0.80–1.15)	0.68	0.70(0.54–0.92)	0.01
rs4946936								
CC	462	44.04	498	47.34	1.00		1.00	
CT	468	44.61	454	43.16	1.11(0.93–1.33)	0.25	1.06(0.81–1.38)	0.69
TT	119	11.34	100	9.51	1.28(0.96–1.72)	0.10	1.38(0.89–2.13)	0.15
CT/TT	587	55.96	554	52.66	1.14(0.96–1.36)	0.13	1.11(0.86–1.43)	0.42
rs4503258								
CC	971	92.56	955	90.78	1.00		1.00	
CT	26	2.48	26	2.47	0.98(0.57–1.71)	0.95	0.66(0.29–1.47)	0.31
TT	52	4.96	71	6.75	0.72(0.50–1.04)	0.08	0.70(0.41–1.20)	0.19
CT/TT	78	7.44	97	9.22	0.79(0.58–1.08)	0.14	0.69(0.44–1.08)	0.10

a: OR and 95%CI without adjusting.

b: Adjusted by logistic regression for age, gender, nationalities, smoking, alcohol intake, chronic HBV infection, and family history of HCC.

### Gene-Environment and Gene-Gene Interaction

The gene-environment interaction analysis revealed that there was evidence of multiplicative interaction between the SNPs (rs17592236, rs4946936 and rs4503258) and environmental risk factors in HCC progression (Table 3). Additionally, an multiplicative interaction between the SNPs rs17592236 and rs4503258 was found in the gene-gene interaction analysis (Table 4).

**Table 3:** Results of *FOXO* gene-environment interaction analyses.

Factors	*β*	SE(*β*i)	*OR*(95% *CI*)^a^	*P-*value
rs17592236×Smoking	0.37	0.10	1.44(1.19~1.76)	<0.01
rs17592236×Alcohol intake	0.26	0.11	1.30(1.06~1.60)	0.01
rs17592236×Chronic HBV infection	1.98	0.08	7.21(6.15~8.45)	<0.01
rs17592236×Family history of HCC	1.11	0.35	3.04(1.53~6.03)	<0.01
rs4946936×Smoking	0.47	0.11	1.60(1.29~1.98)	<0.01
rs4946936×Alcohol intake	0.29	0.11	1.34(1.08~1.67)	0.01
rs4946936×Chronic HBV infection	2.16	0.09	8.68(7.25~10.39)	<0.01
rs4946936×Family history of HCC	1.12	0.36	3.06(1.51~6.22)	0.02
rs4503258×Smoking	-0.84	0.47	1.76(1.28~2.42)	<0.01
rs4503258×Alcohol intake	0.38	0.17	1.46(1.06~2.03)	0.02
rs4503258×Chronic HBV infection	3.36	0.13	28.66(22.20~7.00)	<0.01
rs4503258×Family history of HCC	1.54	0.51	4.67(1.73~12.62)	0.02

a: OR and 95% *CI* for interaction of the cross-product term, adjusted by logistic regression for age, gender, nationalities, smoking, alcohol intake, chronic HBV infection, and family history of HCC.

**Table 4:** Results of gene-gene interaction analyses.

Factors	*β*	SE(*β*i)	*OR*(95% *CI*)^a^	*P-*value
rs17592236×rs4946936	-0.04	0.06	0.96(0.86~1.08)	0.49
rs17592236×rs4503258	-0.28	0.09	0.76(0.63~0.91)	<0.01
rs4946936×rs4503258	-0.04	0.09	0.96(0.80~1.15)	0.65

a: OR and 95% *CI* for interaction of the cross-product term, adjusted by logistic regression for age, gender, nationalities, smoking, alcohol intake, chronic HBV infection, and family history of HCC.

### Luciferase Assays

**Fig 1:**
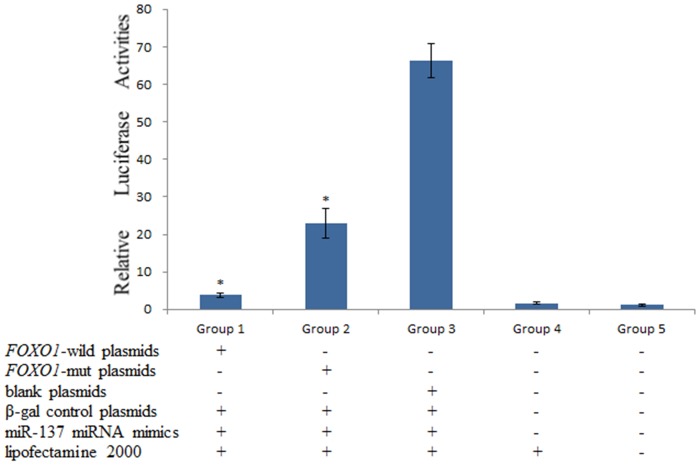
Validation of miR-137 binding site using luciferase assays. Results are represented as mean ± SE. The significance of differences was evaluated by using the t-test (*P<0.001). 293T cells were co-transfected with miR-137 mimics and luciferase reporter constructs carrying *FOXO1* 3’UTR or rs17592236 mutated *FOXO1* 3’UTR fragment. Blank luciferase reporter plasmids were used as a control. The result of luciferase assays indicated that the expression of *FOXO1*-wild constructs was suppressed by miR-137, which was recovered when the potential miR-137 binding site rs17592236 was mutated.

To functionally test our hypothesis that sequence variations in rs17592236 lead to varied miRNA binding affinity and subsequent variations in mRNA degradation, we used a well-established luciferase reporter system as readout for miRNA activity. We cloned a 3385bp fragment from FOXO1 3’UTR containing SNP rs17592236 into miRNA Expression Reporter Vector. Coexpression of miR-137 will subject this reporter mRNA to regulation by miR-137. Thus in this experimental setting, the relative luciferase activity correlates with reporter mRNA stability: higher luciferase activity corresponds to weaker miRNAs affinity to reporter mRNA and vice versa. A total of 5 experimental groups, each containing 6 wells were included in this experiment (Fig. 1). Group 1 and 2 cells were cotransfected hsa-miR-137 miRNA mimics (Ambion) along with the *FOXO1*-wild and *FOXO1*-mut plasmids, respectively. Group 3 cells received pMIR-REPORT plasmid and hsa-miR-137 miRNA mimics, whereas Group 4 cells received only transfection reagents and Group 5 cells received no transfection. As shown in Fig. 1, strongest relative luciferase activity was observed in Group 3 cells. Importantly, Group 2 cells showed higher luciferase activity than Group 1 cells, suggesting that mutation in rs17592236 affected the binding affinity of miR-137 to 3’UTR in messenger RNA (mRNA) of *FOXO1*.

## Discussion

The *FOXO* family members such as *FOXO1*, *FOXO3* and *FOXO4* represent one of several effector arms of PI3K-Akt signaling, which regulates cell proliferation and survival [23]. PI3K-Akt signaling is known to play an important role in the suppression of tumorigenesis [24]. It was proposed that dysregulation of PI3K-Akt activity in human tumors is frequently achieved through genetic alterations of its many signaling components [8]. In the present study, we selected three SNPs within non-coding regions of the *FOXO* genes and found that polymorphism of rs17592236 in *FOXO1* was associated with a reduced risk of HCC. Our results indicate that *FOXO*s may be involved in the development of HCC, consistent with previous studies, showing associations between abnormal expression and localization of *FOXO1* and *FOXO3a* in patients with HCC [12–15].

*FOXO1* is located in chromosome 13q14.1 and encodes 655 amino acids. It is largely expressed in adipose tissue as well as liver tissue [25]. *FOXO1* is an important transcription factor downstream of the PI3K-Akt signaling pathway and has been implicated as a tumor suppressor in several cancers, including mesothelioma [26], Ewing’s sarcoma [27], gastric cancer [28], oral cancer [29], prostate cancer [30] and breast cancer [31]. Its function as a tumor suppressor was thought to be carried out through regulating cell cycle and apoptosis factor expression. Such factors include p21 ^Cip1^, FasL, p27^Kipl^, Cyclin G2, Cyclin D1 and CyclinD2, etc [32]. Interestingly these factors also serve as effectors of the P13k-Akt signaling pathway [33]. Lee et al. [13] found that depleting of Aurora A kinase in HCC cells led to increased *FOXO1* expression and cell cycle arrest. Altogether, existing evidence suggest a PI3K-Akt-*FOXO1* axis that may play a pivotal role in HCC development.

*FOXO1* has been shown to be a target of regulation by many cancer-related miRNAs. These include miR-27a, miR-96, miR-182 in breast cancer [31], miR-196a in cervical cancer [34], and miR-223 in colorectal cancer, cervical cancer, and HCC [35]. Here we have demonstrated that a mutation in rs17592236 may decrease the affinity for miR-137 to bind to the 3’UTR of *FOXO1* mRNA and consequently reduce miR-137-mediated inhibition of *FOXO1* expression. MiR-137 is located on human chromosome 1p22 and has been suggested to function as a tumor suppressor via cell cycle control in several cancers including colorectal cancer [36], squamous cell carcinoma [37] and melanoma [38]. Additionally, miR-137 has been suggested to be involved in HCC [39], although the underlying mechanism was not clearly understood. The present study provides evidence for a model in which miR-137 can modulate *FOXO1* expression through SNP rs17592236 and thereby affecting the PI3K-Akt-*FOXO1* axis in HCC carcinogenesis.

HCC is a complex disease caused by a combination of hereditary and environmental factors [1]. Gene-environment and gene-gene interactions were implicated in HCC development [40, 41]. Consistent with previous studies, the current study also demonstrated genetic interactions between the SNPs as well as interactions with environmental risk factors such as chronic HBV infection, smoking and alcohol intake, leading to enhanced HCC development. These data lend support to the relevance of our findings to in vivo HCC carcinogenesis.

To summarize, we selected three SNPs within non-coding regions of *FOXO*s and observed that polymorphism of rs17592236 in *FOXO1* was associated with reduced risk of HCC. This was most likely due to enhanced binding of has-miR-137 to the 3’UTR of *FOXO1* mRNA. These findings support the hypothesis that sequence variations of SNPs at miRNA target sites may be associated with risk of HCC, by affecting miRNA binding affinity and subsequent degradation of *FOXO* mRNAs.- This information may be important for clinical diagnosis and for future prevention and targeted therapy of HCC. Further research is needed to elucidate how miR-137 regulates the PI3K-Akt-*FOXO1* axis in vivo and in vitro at the molecular level.

## References

[1] Farazi PA, DePinho RA. Hepatocellular carcinoma pathogenesis: from genes to environment. Nature Reviews Cancer. 2006; 6: 674–687. 16929323 10.1038/nrc1934

[2] Calnan DR, Brunet A. The FoxO code. Oncogene. 2008; 27: 2276–2288. 10.1038/onc.2008.21 18391970

[3] Alikhani M, Alikhani Z, Graves DT. FOXO1 functions as a master switch that regulates gene expression necessary for tumor necrosis factor-induced fibroblast apoptosis. Journal of Biological Chemistry. 2005; 280: 12096–12102. 15632117 10.1074/jbc.M412171200

[4] Ward EC, Hoekstra AV, Blok LJ, Hanifi-Moghaddam P, Lurain JR, Singh DK, et al The regulation and function of the forkhead transcription factor, Forkhead box O1, is dependent on the progesterone receptor in endometrial carcinoma. Endocrinology. 2008; 149: 1942–1950. 18096667 10.1210/en.2007-0756 PMC2276720

[5] Accili D, Arden KC. FoxOs at the crossroads of cellular metabolism, differentiation, and transformation. Cell. 2004; 117: 421–426. 15137936 10.1016/s0092-8674(04)00452-0

[6] Maiese K, Chong ZZ, Shang YC, Hou J. A “FOXO” in sight: targeting Foxo proteins from conception to cancer. Medicinal research reviews. 2009; 29: 395–418. 10.1002/med.20139 18985696 PMC2666780

[7] Trotman LC, Alimonti A, Scaglioni PP, Koutcher JA, Cordon-Cardo C, Pandolfi PP. Identification of a tumour suppressor network opposing nuclear Akt function. Nature. 2006; 441: 523–527. 16680151 10.1038/nature04809 PMC1976603

[8] Paik J-H, Kollipara R, Chu G, Ji H, Xiao Y, Ding Z, et al FoxOs are lineage-restricted redundant tumor suppressors and regulate endothelial cell homeostasis. Cell. 2007; 128: 309–323. 17254969 10.1016/j.cell.2006.12.029 PMC1855089

[9] Arden KC. FoxOs in tumor suppression and stem cell maintenance. Cell. 2007; 128: 235–237. 17254960 10.1016/j.cell.2007.01.009

[10] Fu Z, Tindall D. FOXOs, cancer and regulation of apoptosis. Oncogene. 2008; 27: 2312–2319. 10.1038/onc.2008.24 18391973 PMC2819403

[11] Greer E, Brunet A. FOXO transcription factors in ageing and cancer. Acta physiologica. 2008; 192: 19–28. 10.1111/j.1748-1716.2007.01780.x 18171426

[12] Fei M, Lu M, Wang Y, Zhao Y, He S, Gao S, et al Arsenic trioxide-induced growth arrest of human hepatocellular carcinoma cells involving FOXO3a expression and localization. Medical Oncology. 2009; 26: 178–185. 10.1007/s12032-008-9105-8 18937079

[13] Lee S-Y, Lee GR, Woo D-H, Park NH, Cha HJ, Moon Y, et al Depletion of Aurora A leads to upregulation of FoxO1 to induce cell cycle arrest in hepatocellular carcinoma cells. Cell Cycle. 2013; 12: 67 23255113 10.4161/cc.22962 PMC3570518

[14] Lu M, Ma J, Xue W, Cheng C, Wang Y, Zhao Y, et al The expression and prognosis of FOXO3a and Skp2 in human hepatocellular carcinoma. Pathology & Oncology Research. 2009; 15: 679–687. 19404778 10.1007/s12253-009-9171-z

[15] Horie Y, Suzuki A, Kataoka E, Sasaki T, Hamada K, Sasaki J, et al Hepatocyte-specific Pten deficiency results in steatohepatitis and hepatocellular carcinomas. Journal of Clinical Investigation. 2004; 113: 1774–1783. 15199412 10.1172/JCI20513 PMC420505

[16] Sethupathy P, Collins FS. MicroRNA target site polymorphisms and human disease. Trends in genetics. 2008; 24: 489–497. 10.1016/j.tig.2008.07.004 18778868

[17] Yu Z, Li Z, Jolicoeur N, Zhang L, Fortin Y, Wang E, et al Aberrant allele frequencies of the SNPs located in microRNA target sites are potentially associated with human cancers. Nucleic acids research. 2007; 35: 4535–4541. 17584784 10.1093/nar/gkm480 PMC1935019

[18] Pezzoli K, Tukey R, Sarabia H, Zaslavsky I, Miranda ML, Suk WA, et al The NIEHS Environmental Health Sciences Data Resource Portal: placing advanced technologies in service to vulnerable communities. Environmental health perspectives. 2007; 115: 564–571. 17450225 10.1289/ehp.9817 PMC1852670

[19] Packer BR, Yeager M, Burdett L, Welch R, Beerman M, Qi L, et al SNP500Cancer: a public resource for sequence validation, assay development, and frequency analysis for genetic variation in candidate genes. Nucleic acids research. 2006; 34: D617–D621. 16381944 10.1093/nar/gkj151 PMC1347513

[20] Betel D, Wilson M, Gabow A, Marks DS, Sander C. The microRNA. org resource: targets and expression. Nucleic acids research. 2008; 36: D149–D153. 18158296 10.1093/nar/gkm995 PMC2238905

[21] Chun HB, Xiao QQ, Xiao YZ, Yan Y, Jin MH, Xue JF. Association of interleukin-10 gene poly morphism with hepatocellular carcinoma. Chinese Journal of Public Health. 2011; 27: 309–311.

[22] Zeng XY, Yu HP, Qiu XQ, Ji L, Li LM. Correlation of polymorphism of DNA repair gene XRCC3 with susceptibility to hepatocellular carcinoma in regions of high HCC incidence rate in Guangxi. Chinese Journal of Cancer Prevention and Treatment. 2009; 16: 1629–1633.

[23] Zhang Y, Gan B, Liu D, Paik JH. FoxO family members in cancer. Cancer biology & therapy. 2011; 12: 253–259. 21613825 10.4161/cbt.12.4.15954

[24] Luo J, Manning BD, Cantley LC. Targeting the PI3K-Akt pathway in human cancer: rationale and promise. Cancer cell. 2003; 4: 257–262. 14585353 10.1016/s1535-6108(03)00248-4

[25] Nakae J, Kitamura T, Kitamura Y, Biggs WH III, Arden KC, Accili D. The forkhead transcription factor Foxo1 regulates adipocyte differentiation. Developmental cell. 2003; 4: 119–129. 12530968 10.1016/s1534-5807(02)00401-x

[26] Tomasetti M, Nocchi L, Staffolani S, Manzella N, Amati M, Goodwin J, et al MicroRNA-126 Suppresses Mesothelioma Malignancy by Targeting IRS1 and Interfering with Mitochondrial Function. Antioxidants & redox signaling. 2014; 21: 2109–2125. 24444362 10.1089/ars.2013.5215 PMC4215384

[27] Niedan S, Kauer M, Aryee D, Kofler R, Schwentner R, Meier A, et al Suppression of FOXO1 is responsible for a growth regulatory repressive transcriptional sub-signature of EWS-FLI1 in Ewing sarcoma. Oncogene. 2013; 33: 3927–3938. 10.1038/onc.2013.361 23995784 PMC4114138

[28] Park J, San Ko Y, Yoon J, Kim MA, Park J-W, Kim WH, et al The forkhead transcription factor FOXO1 mediates cisplatin resistance in gastric cancer cells by activating phosphoinositide 3-kinase/Akt pathway. Gastric Cancer. 2013; 17: 1–8. 10.1007/s10120-013-0252-z 24202965

[29] Huang CY, Chan CY, Chou IT, Lien CH, Hung HC, Lee MF, et al Quercetin induces growth arrest through activation of FOXO1 transcription factor in EGFR-overexpressing oral cancer cells. The Journal of nutritional biochemistry. 2013; 24: 1596–1603. 10.1016/j.jnutbio.2013.01.010 23618529

[30] Fendler A, Jung M, Stephan C, Erbersdobler A, Jung K, Yousef GM, et al The Antiapoptotic Function of miR-96 in Prostate Cancer by Inhibition of FOXO1. PloS one. 2013; 8: e80807 10.1371/journal.pone.0080807 24260486 PMC3834337

[31] Guttilla IK, White BA. Coordinate regulation of FOXO1 by miR-27a, miR-96, and miR-182 in breast cancer cells. J Biol Chem. 2009; 284: 23204–23216. 10.1074/jbc.M109.031427 19574223 PMC2749094

[32] Furukawa-Hibi Y, Kobayashi Y, Chen C, Motoyama N. FOXO transcription factors in cell-cycle regulation and the response to oxidative stress. Antioxidants & redox signaling. 2005; 7: 752–760. 15890021 10.1089/ars.2005.7.752

[33] Jones SM. Connecting signaling and cell cycle progression in growth factor-stimulated cells. Oncogene. 2000; 19:5558–67. 11114735 10.1038/sj.onc.1203858

[34] Hou T, Ou J, Zhao X, Huang X, Huang Y, Zhang Y. MicroRNA-196a promotes cervical cancer proliferation through the regulation of FOXO1 and p27(Kip1). Br J Cancer. 2014; 110: 1260–1268. 10.1038/bjc.2013.829 24423924 PMC3950858

[35] Wu L, Li H, Jia CY, Cheng W, Yu M, Peng M, et al MicroRNA-223 regulates FOXO1 expression and cell proliferation. FEBS Lett. 2012; 586: 1038–1043. 10.1016/j.febslet.2012.02.050 22569260

[36] Liang L, Li X, Zhang X, Lv Z, He G, Zhao W, et al MicroRNA-137, an HMGA1 target, suppresses colorectal cancer cell invasion and metastasis in mice by directly targeting FMNL2. Gastroenterology. 2013; 144: 624–635. 10.1053/j.gastro.2012.11.033 23201162

[37] Langevin SM, Stone RA, Bunker CH, Lyons-Weiler MA, LaFramboise WA, Kelly L, et al MicroRNA-137 promoter methylation is associated with poorer overall survival in patients with squamous cell carcinoma of the head and neck. Cancer. 2011; 117: 1454–1462. 10.1002/cncr.25689 21425146 PMC3117118

[38] Bemis LT, Chen R, Amato CM, Classen EH, Robinson SE, Coffey DG, et al MicroRNA-137 targets microphthalmia-associated transcription factor in melanoma cell lines. Cancer Res. 2008; 68: 1362–1368. 10.1158/0008-5472.CAN-07-2912 18316599

[39] Yang J, Zhou F, Xu T, Deng H, Ge Y, Zhang C, et al Analysis of sequence variations in 59 microRNAs in hepatocellular carcinomas. Mutation Research/Fundamental and Molecular Mechanisms of Mutagenesis. 2008; 638: 205–209. 17900631 10.1016/j.mrfmmm.2007.08.007

[40] Chen CJ, Chen DS. Interaction of hepatitis B virus, chemical carcinogen, and genetic susceptibility: multistage hepatocarcinogenesis with multifactorial etiology. Hepatology. 2002; 36: 1046–1049. 12395312 10.1053/jhep.2002.37084

[41] Yu MW, Yang SY, Chiu YH, Chiang YC, Liaw YF, Chen CJ. Ap53genetic polymorphism as a modulator of hepatocellular carcinoma risk in relation to chronic liver disease, familial tendency, and cigarette smoking in hepatitis B carriers. Hepatology. 1999; 29: 697–702. 10051470 10.1002/hep.510290330

